# Building university-based boundary organisations that facilitate impacts on environmental policy and practice

**DOI:** 10.1371/journal.pone.0203752

**Published:** 2018-09-13

**Authors:** Christopher Cvitanovic, Marie F. Löf, Albert V. Norström, Mark S. Reed

**Affiliations:** 1 Centre for Marine Socioecology, University of Tasmania, Hobart, Australia; 2 CSIRO Oceans & Atmosphere, Hobart, Australia; 3 Baltic Sea Centre, Stockholm University, Stockholm, Sweden; 4 Stockholm Resilience Centre, Stockholm University, Stockholm, Sweden; 5 Centre for Rural Economy and Institute for Agri-Food Research and Innovation, School of Natural and Environmental Sciences, Newcastle University, Newcastle, United Kingdom; Universidad de Castilla-La Mancha, SPAIN

## Abstract

Responding to modern day environmental challenges for societal well-being and prosperity necessitates the integration of science into policy and practice. This has spurred the development of novel institutional structures among research organisations aimed at enhancing the impact of environmental science on policy and practice. However, such initiatives are seldom evaluated and even in cases where evaluations are undertaken, the results are rarely made publicly available. As such there is very little empirically grounded guidance available to inform other organisations in this regard. To help address this, the aim of this study is to evaluate the Baltic Eye Project at Stockholm University–a unique team consisting of researchers from different fields, science communicators, journalists and policy analysts–working collectively to support evidence-informed decision-making relating to the sustainable management of the Baltic Sea environment. Specifically, through qualitative interviews, we (1) identify the impacts achieved by the Baltic Eye Project; (2) understand the challenges and barriers experienced throughout the Baltic Eye Project; and (3) highlight the key features that are needed within research organisations to enhance the impact of science on policy and practice. Results show that despite only operating for three years, the Baltic Eye Project has achieved demonstrable impacts on a range of levels: impacts on policy and practice, impacts to individuals working within the organisation and impacts to the broader University. We also identify a range of barriers that have limited impacts to date, such as a lack of clear goals at the establishment of the Baltic Eye Project and existing metrics of academic impact (e.g. number of publications). Finally, based on the experiences of employees at the Baltic Eye Project, we identify the key organisational, individual, financial, material, practical, political, and social features of university-based boundary organisations that have impact on policy and practice. In doing so this paper provides empirically-derived guidance to help other research organisations increase their capacity to achieve tangible impacts on environmental policy and practice.

## Introduction

Successfully navigating modern day environmental challenges requires the integration of new and evolving knowledge into decision-making processes [1]. Science is one form of knowledge that is critical in this regard [2], however, the uptake and integration of scientific research into decision-making processes remains a significant challenge [3–5]. For this reason an emergent body of literature has sought to identify the barriers preventing the use of environmental science by decision-makers, and in turn, develop strategies for improving the extent to which environmental science informs policy and practice [6, 7]. A recurrent theme throughout this literature has been the need for institutional innovation to support and facilitate the active and real-time exchange of knowledge between environmental scientists and practitioners to enhance the impact of environmental science on policy and practice [8–11].

In recognition of this need, new and innovative institutional structures have been developed and implemented to enhance the use of environmental science in policy and practice. For example, it has become increasingly common for decision-making organisations to ‘embed’ scientists within their organisations [12–14]. There is evidence that this increases the likelihood that priority knowledge gaps will be answered and outcomes will be shared quickly throughout the decision-making agency, thus improving the likelihood that the information will inform decision-making processes and deliver beneficial impacts [15]. Similarly, research institutions have sought to adopt new institutional structures that actively facilitate solutions-driven and transdisciplinary collaborations with policy-makers and other societal actors to enhance the use of science in decision-making processes [16, 17]. There has also been the emergence of new organisational roles and functions such as knowledge brokers [18, 19], which focus specifically on improving two-way knowledge exchange among environmental scientists and decision-makers to support evidence-informed decision-making [20–22].

Despite the increased implementation of new institutional structures to enhance the impact of environmental science on policy and practice, such initiatives are seldom evaluated. This is, in part, because of the well-documented difficulties associated with defining and measuring specific impacts and outcomes that occur at the interface of science, policy and practice, which are often not readily quantifiable [23]. For example, it is increasingly clear that the relationship between science and impact is often indirect, non-linear, complex and uncertain [24, 25]. Undertaking such evaluations, however, is necessary for the identification of the factors that limit and/or enable the efficacy of such initiatives [26, 27]. This, in turn, can provide empirically grounded guidance to assist other organisations seeking to develop and implement new institutional approaches to increase their impact at the science-policy-practice interface for better environmental outcomes [28].

This study seeks to contribute towards filling this gap, by identifying the features of university-based boundary organisations that enable and/or limit impact at the science-policy-practice interface. We do this via an in-depth analysis of the Baltic Eye Project at Stockholm University. The Baltic Eye Project was established in 2013 within the Baltic Sea Centre at Stockholm University with the core goal of supporting evidence-informed decision-making on issues relating to the management of the Baltic Sea environment. To achieve this goal the Baltic Eye Project has developed a unique team consisting of researchers from different fields (e.g. ecology, ecotoxicology, environmental chemistry, biogeochemistry, agronomy, and oceanography), science communicators, journalists and policy analysts–all of whom are co-located in a single location to promote high levels of integration among all aspects of their work.

There are three specific aims with this evaluation of the Baltic Eye Project; (1) identify the impacts achieved by the Baltic Eye Project; (2) understand the challenges and barriers experienced throughout the Baltic Eye Project; and (3) elucidate the key features that are needed within research organisations to enhance the impact of science on policy and practice. While we acknowledge that the interface between environmental science, policy and practice is highly context-specific across cultures, space and time, focusing on a single initiative in this manner allows for the in-depth exploration of the study objectives. In turn, this allows for the generation of a set of key design features to help other university-based boundary organisations to understand how to build capacity to operate more effectively at the science-policy-practice interface.

## Methods

This study has been approved by the Tasmanian Social Sciences Human Research Ethics Committee (Approval reference: TSSHREC: H0016870).

### Case study: The Baltic Eye Project at Stockholm University

The Baltic Sea is one of the largest bodies of brackish water on the planet, supporting both marine and freshwater species. It is bordered by nine countries–Sweden, Denmark, Estonia, Finland, Germany, Latvia, Lithuania, Poland and Russia–with approximately 85 million people living directly within the drainage area. Of these, approximately 15 million people live in the coastal margins directly adjacent to the Sea [29]. The Baltic Sea provides critical ecosystems goods and services that directly underpin the prosperity and well-being of local communities, for example, via the provision of food, employment, economic stimulus through tourism, and recreational benefits [30–32].

However, human activities throughout the region have placed the Baltic Sea under increased pressure and have compromised its ability to provide ecosystem goods and services for human well-being. For example, eutrophication driven by excessive inputs of nutrients into the Baltic Sea from activities such as intensive farming, in combination with the naturally low levels of water exchange, has led to reduced water clarity, the expansion of anoxic areas, altered species composition, and increased growth of toxic algal blooms [33–37]. The economic damages associated with eutrophication throughout the Baltic Sea region are estimated to be 3.8–4.4 billion euros annually [32]. Eutrophication, however, is only one threat to the Baltic Sea and others include (but are not limited to) the introduction of hazardous substances and marine litter (e.g. microplastics) [38], changes to salinity and water temperature [39] and overfishing [40]. The combined impacts of these threats have, and will continue to, significantly alter the functioning of the Baltic Sea with potential adverse effects on the well-being and prosperity of millions of people that directly depend on the goods and services it provides [33, 41].

The numerous, and interacting, environmental stressors acting on the Baltic Sea present a significant challenge to decision-makers, particularly given their transboundary nature (a comprehensive review of the current status of the Baltic Sea and the interacting stressors can be found in [29, 32 and 41]). To this end, in 2013 Stockholm University established the Baltic Eye Project, a dedicated team focused solely on supporting evidence-based decision-making in relation to the management of the Baltic Sea environment. The Baltic Eye project is embedded in the larger Stockholm University Baltic Sea Centre, whose mission is to strengthen marine research and education at Stockholm University, provide infrastructure for this research and collect useful knowledge and communicate it to diverse societal actors.

Starting with only a small team of researchers and a single communications officer in mid-2014, the Baltic Eye Project team’s structure and composition has evolved as the group have learnt to navigate the boundary between science, policy and practice in relation to the management of the Baltic Sea environment. At the time of this study the team was comprised of 17 individuals, including a team of scientists (ecotoxicology, biogeochemistry, ecology, environmental chemistry, oceanography and agronomy), policy analysts, science communicators, a journalist and a technician. While on their own many of these functions exist in many research organisations, they are seldom integrated into a single team in this way, and focused on a specific issue as is the case for the Baltic Eye Project (e.g. communication officers typically provide broad support for large departments opposed to specialised teams). Furthermore, the ratio between research and non-research staff is balanced in the Baltic Eye, as opposed to being highly skewed towards research, which is the norm in most research organisations. Thus, the Baltic Eye Project represents a unique opportunity to understand how to build research organisations to facilitate greater impacts on policy and practice, so that other research organisations can learn from their experiences and understand how to build capacity in this regard.

### Data collection

To address the three objectives of this research we used a qualitative research approach, conducting semi-structured interviews of participants between November and December 2017. Qualitative methods were selected to allow for a comprehensive and in-depth exploration of the issues relating to the study objectives [42]. These methods also allow us to generate a set of key design features to guide the efforts of other research institutions seeking to enhance their impact at the interface of science, policy and practice [43, 44].

Interviews were completed with 16 of the 17 staff employed within the Baltic Eye Project, thus representing 94% of our focal sample group. The remaining individual was not available to participate at the time that interviews were conducted. All interviews were undertaken by a single member of the research team (CC) to ensure a consistent approach, and were guided by a set of questions (i.e. an interview guide) that were designed to directly explore the perceptions of participants against each of the three study objectives (full interview guide at S1 Appendix). The interview guide was developed by drawing on and adapting existing published evaluations of knowledge exchange activities, tailored for the purpose of this study [22, 28, 43]. To ensure the utility of the interview guide it was first pilot tested among the research team, refined accordingly and then tested with two members of the Baltic Eye Project. While in some circumstances it is not appropriate to test the interview guide on individuals who are part of the focal research group [42], the specific nature of our case study made it important to ensure that the questions were directly understandable and applicable to the specific context [28]. Further, in this instance it was important to test the interview guide in this manner as all interviews were conducted in English (the working language within the Baltic Eye Project), although English was not the native language for most participants. Thus, ensuring clarity in the interview guide was critical. Following the piloting process, no major changes to the interview guide were required, however, the wording of some questions were revised slightly for clarity.

Prior to commencing each interview, the purpose of the research was explained to the participant and formal written consent to participate was obtained (in accordance with Human Research Ethics Procedure TSSHREC: H0016870). The majority of interviews were undertaken face-to-face, however five were undertaken via Skype. Interviews lasted between 23–95 minutes, but more typically were between 45–60 minutes in duration. All interviews were audio recorded and professionally transcribed to ensure accuracy of the transcript.

### Data analysis

All interview transcripts were analysed using NVIVO 10 qualitative data analysis software. The analysis consisted of broad thematic coding against the research objectives:

1. What impacts have been achieved to date by the Baltic Eye Project;
2. What barriers have been experienced in the Baltic Eye Project; and
3. What are the critical features that are needed within research teams to enhance the impact of environmental science on policy and practice.

While the research objectives formed the basis of the coding, analysis of the raw data was completed following an inductive approach, based on Grounded Theory Analysis [45], so that the research findings could emerge from the interviews without the restraints imposed by structured methodologies [46]. In doing so, each individual response was coded against a set of descriptors designed to identify emergent themes and to capture the key elements of these themes [42]. Further, to ensure that emerging themes were valid and relevant, the evolving interpretations were continually verified against the raw data from which they were derived (following previous studies including [47, 48]).

## Results

The interview coding produced 19 themes that mapped to each of the three research objectives (Table 1). While some overlap among themes is observed due to the chosen methodology, this approach allowed for the identification of (1) the key impacts arising from the Baltic Eye Project; (2) the barriers and challenges experienced in the Baltic Eye Project; and (3) the features and capacities that are critical for inclusion when building research organisations that aim to enhance the impact of environmental science on policy and practice. While an analysis hierarchy of key themes is presented here to provide an overview of the coding results (Table 1), it should be noted that the frequency simply refers to the number of times each theme was mentioned by participants, rather than the level of importance participants placed on the specific issue. To this end, specific themes within each research objectives are addressed in the following sub-sections.

**Table 1:** Analysis hierarchy of themes derived from interviews with research participants. Frequency is the number of times a theme was coded across all interviews, while the number of sources represents the number of unique interviewees who raised the theme (total interviewees = 16).

Research Objective	Theme	Frequency	Number of sources
1. Impacts achieved by the Baltic Eye Project	a. Impacts on policy and decision-making	34	15
	b. Personal impacts	36	15
	c. University wide impacts	23	13
2. Barriers experienced in the Baltic Eye Project	a. Lack of direction/goals	22	12
	b. Current metrics of science impact	14	10
	c. Novelty (no existing blueprint)	10	10
	d. Spread too widely (too much to do)	11	9
	e. University culture	10	7
	f. Lack of policy expertise from the start	4	4
	g. Current structure	4	3
	Making science understandable	3	2
	i. Disciplinary differences within team	2	2
3. Capacities and attributes that enhance impact between science, policy and practice	a. Organisational	145	16
	b. Individual	61	16
	c. Financial	29	13
	d. Material	19	9
	e. Practical	11	10
	f. Political	10	9
	g. Social	7	5

### Impacts achieved by the Baltic Eye Project

The interview analysis produced three themes relating to the impacts achieved by the Baltic Eye Project: impacts on policy and practice, individual impacts (i.e. impacts to individuals working within the Baltic Eye Project), and impacts to the broader University (Table 1). In relation to impacts on policy and decision-making, all participants noted the challenges associated with measuring specific impacts on policy (e.g. adoption of specific recommendations into policy) and the time required to achieve impacts (noting that the team had only been established for three years). Irrespective, participants did identify a number of specific instances that they believed demonstrated that they were having an impact on policy in relation to the management of the Baltic Sea environment: *‘…we have been successful in influencing some policy processes…in relation to microplastics*, *and coastal management as well’* (ID 6). For example, eight participants highlighted how information provided by the Baltic Eye Project to decision-makers (e.g. in policy briefs), was reflected in official speeches and documents: *‘…we can clearly see that decision-makers and local Swedish Ministers are using the information that we supply in their speeches…they are phrasing things the same way that we are phrasing them’* (ID 7). This was reiterated by another participant who stated that: *‘…when the government launched their prioritised areas for funding action…you could really identify our thematic areas…you could identify exactly the punchline from our policy briefs’* (ID 16).

In addition, participants outlined numerous occasions where Swedish politicians or decision-makers had publicly acknowledged the contribution of information provided to them by the Baltic Eye Project. As explained by one participant: *‘The Minister for the Environment praised us publicly at this big meeting of decision-makers…and thanked us for the information and support that we had provided in preparation for their meeting in New York in relation to SDG 14* [Sustainable Development Goal—Life below water]’ (ID 8). Participants also spoke about how they felt that the Baltic Eye Project had become a trusted advisor for Swedish decision-makers: ‘…[In Sweden] *politicians reach out to us now*, *proactively with questions…they come to us with questions about the Baltic Sea’* (ID 12).

Participants also identified a number of impacts that working within the Baltic Eye Project has had on them as individuals (Table 1). The most commonly discussed individual impact was increased job satisfaction (identified by 13 participants). This was largely driven by the learning opportunities provided to individuals from working within a diverse team (e.g. development of new skills): *‘I learn so much*, *so much*, *every day*. *I learn about how science works*, *how research processes work*, *how to communicate science better…’* (ID 13). Closely related to this, participants also spoke about how working within the Baltic Eye Project had led to a greater sense of personal achievement, as they could see how the science that was been generated was having an actual impact on policy and practice. Finally, participants also stated that working within the Baltic Eye Project had a personal impact by helping team members to establish new and broader social networks, as highlighted by one scientist from the team: *‘You get a totally different kind of network that you don’t get working in regular scientific positions’* (ID 12).

Finally, participants also outlined a number of ways in which they felt the Baltic Eye Project had achieved an impact on the broader university in which it is situated (Stockholm University) (Table 1). Specifically, participants spoke about how the Baltic Eye Project was able to serve as a role model for other area scientists and research groups within the university, and how they have been able to provide them with guidance and advice in a number of areas relating to the interface of science, policy and practice: *‘We are exposing Stockholm University researchers a bit more to policy…helping them to understand the societal relevance of their research’* (ID 5). Interview coding also revealed that participants felt that the proactive approach taken within the Baltic Eye Project in relation to communication and stakeholder engagement had raised the overall profile of the university, and its various institutions, to external stakeholders.

### Barriers to achieving impact experienced in the Baltic Eye Project

Despite the successes achieved to date, participants also outlined several barriers that they felt had limited the overall impact of the Baltic Eye Project (Table 1). The most significant of these, identified by 12 of the 16 participants, was the lack of a clear direction and goals at the commencement of the Baltic Eye Project: *‘…in the beginning we didn’t have enough direction…we didn’t know what type of organisation we were meant to be…this caused insecurity as we were not sure what was expected of us’* (ID11). Several participants also noted how the lack of a strategic direction within the Baltic Eye Project made it difficult to know which activities to prioritise, leading to minor conflict among team members in the beginning: *‘…it became like a competition between the team*, *with everyone trying to show that they were really active in a lot of areas’* (ID6).

Closely related to this, participants also commonly discussed the novelty of the Baltic Eye Project and how there was no blueprint to guide their initial efforts and activities, which limited their ability to establish a strategic direction on commencement (Table 1): *‘I think the main challenge has been that there is no really good blueprint…it’s been very much trial and error’* (ID 2). This was supported by another participant who said: *‘…we are starting something new and don’t really know how to do it…we need to find ways of working that are really successful’* (ID 14). Participants outlined how this led to an iterative approach to working, which in combination with the need to be reactive to policy, has led to a situation whereby many team members feel as though their efforts are spread too widely and that they are doing too much: *‘You can’t have one person doing all those things…internally it is wearing people down’* (ID 13).

Other commonly discussed barriers related to the ways in which impact is measured in scientific institutions, and specifically the ‘publish or perish’ culture of academia (Table 1). For example, ten out of the 16 participants outlined how traditional metrics of science impact (e.g. number of publications, number of citations, impact factor of journals, etc.) were not applicable to members of the Baltic Eye Project, given that their primary function was not producing new scientific knowledge, but rather synthesising the existing literature to support evidence-informed decision-making. As a result, members of the Baltic Eye Project expressed concern about how this might impact their career longevity and progression, as illustrated by this participant: *‘I am giving up my academic career to work here…because I am not going to publish as much…I will not become a professor…and what happens for me after the* [Baltic Eye Project] *is unclear…but it won’t be in academia’* (ID 4). Building on this, seven participants (Table 1) noted how the novel approach of the Baltic Eye Project, whereby real world impacts are prioritised over publications, made it challenging for team members to integrate into the broader university system and culture: *‘…we are building something new…we don’t have to adjust to an old hierarchical culture…we need to find* [new] *ways to integrate this idea into the old university culture’* (ID 14).

### Features of university-based research organisations that enhance impact on policy and practice

**Fig 1:**
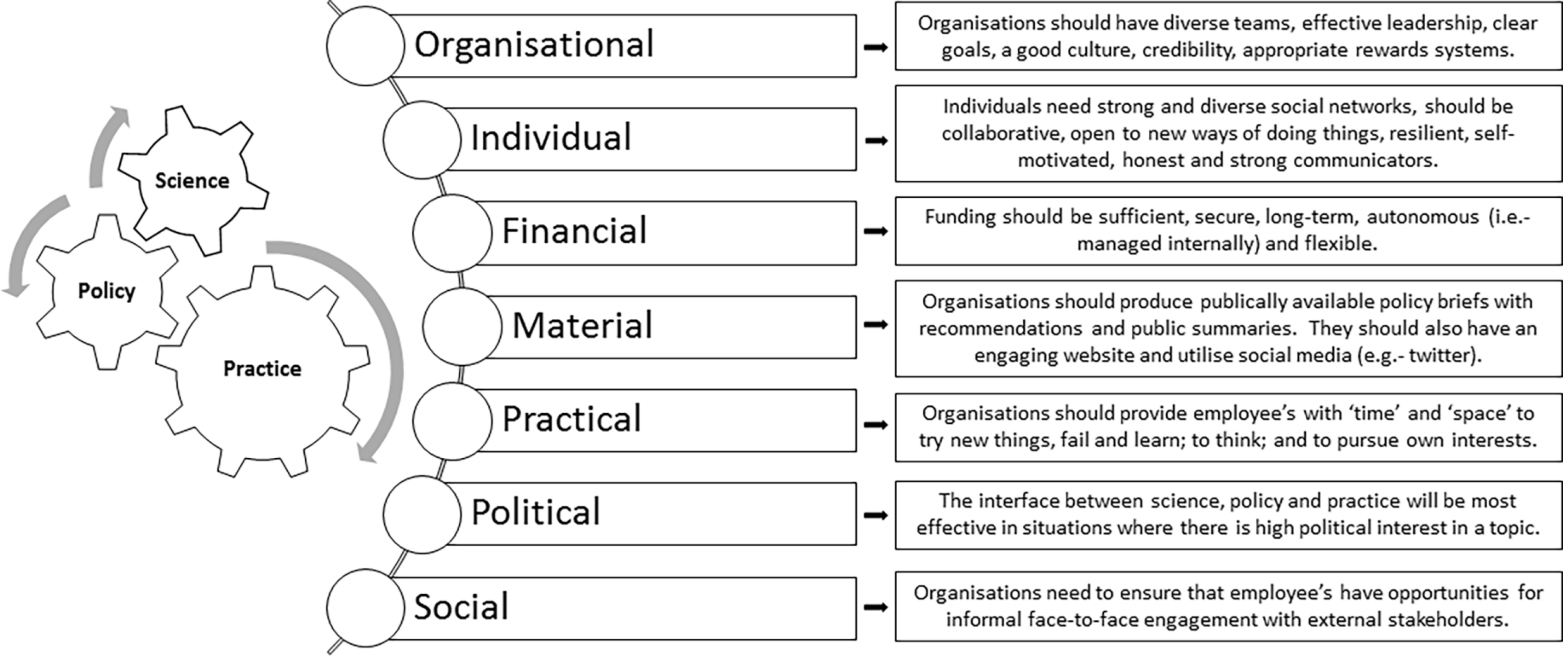
Features of university-based boundary organisations that have impact on policy and practice.

The interview analysis identified seven themes relating to the features of research organisations that aim to enhance the impact of environmental science on policy and practice: (i) Organisational, (ii) Individual, (iii) Financial, (iv) Material, (v) Practical, (vi) Political and (vii) Social (Table 1). Of these, organisational features were most frequently discussed, which is not surprising given the focus of this study. However, individual capacities and financial capacities were also discussed by 16 and 13 of the study participants, respectively. In this section, we draw upon the experiences and learnings of study participants and outline the critical features and capacities of research organisations that support an effective relationship between environmental science, policy and practice, so as to provide a blueprint for other organisations seeking to achieve more impact at this interface (Fig 1).

At the organisational level, participants identified eight features that are necessary to augment the impact science has on policy and practice (Fig 1). Of these, the most commonly discussed was the need for a diversity of skills and expertise among team members: *‘…you need to have people with different backgrounds who can draw upon their backgrounds and contribute their different knowledge bases…*’ (ID 5). In particular, participants discussed how the greatest impacts were achieved in the Baltic Eye Project when scientists, policy-analysts, science-communicators and journalists were co-located in a single team. Participants also commonly identified the presence of effective leaders as a key organisational feature (Fig 1). Specifically, participants discussed the importance of having engaged, supportive and strategic leaders who helped to establish clear priorities in support of the broader objectives of the team. Participants also spoke about the need for leaders to have diverse backgrounds and experiences across both science and policy, so as to have a comprehensive knowledge of both worlds. The interview coding also revealed that institutional credibility is a key feature of research organisations that want to have an impact on policy and practice (Fig 1), and that this is best achieved via honest brokerage opposed to advocacy for a single issue.

Other organisational features discussed by participants included the need for clear goals and a good team culture (Fig 1). With respect to goals, participants spoke about the need for goals to be established and agreed jointly among all team members so that they account for the variety of roles within the team: ‘*If goals are formulated together then everyone feels ownership over the goals…it energises them towards* [achieving the] *goals’* (ID 14). Participants also spoke about the need for goals to be ambitious, strategic and measurable so that progress can be monitored and assessed over time. Having a good team culture was also identified as a critical feature, particularly given the novelty of the Baltic Eye Project, as a good culture gives people with the confidence to experiment and try new approaches for impacting policy, without the fear of been judged or criticised by their colleagues. In this respect, several participants spoke about how an organisation’s culture is largely determined by the leadership team, who should seek to create a safe and respectful environment for their employees. Finally, based on their experiences working in the Baltic Eye Project, participants identified the need for research organisations to develop new metrics of ‘science impact’ that account for the diversity of functions undertaken through such initiatives (i.e. not simply judging scientists by their publications), new career pathways that legitimise the type of work undertaken through such initiatives and allow for career progression, and new training programs to diversify the skill-set of scientists (particularly in relation to generating research impact on policy and practice) (Fig 1).

In addition to the organisational features outlined above, study participants also identified several individual (i.e. personal) attributes that are necessary among team members to support a more effective relationship between science, policy and practice (Fig 1). In particular, the interview coding revealed that any individual within research organisations seeking to have impact on policy and practice should have strong and diverse stakeholder networks and be willing to work collaboratively with all stakeholders: *‘I have come to believe that personal relationships are really important…whether it is a relationship with a journalist…or a politician…when you trust them and they trust you*, *you have the best chance of understanding one another* [and working together]’ (ID 15). Participants also reflected on the challenges on working at the interface of science, policy and practice (as detailed in the ‘Barriers’ section above) and emphasised the importance of having individuals who are open to new ways of doing things (and willing to learn new things), resilient (i.e. demonstrates perseverance through difficult situations) and self-motivated. Finally, the analysis identified the importance of individuals within research teams having strong skills in communication and outreach.

The interview analysis also elucidated several financial features that can help research organisations achieve more impact at the interface of science, policy and practice (Fig 1). In relation to financial features, participants discussed the importance of having secure and long-term funding (at least 5 years). This was identified as necessary given the time needed to develop relationships among team members and other stakeholders, and the long time-scales in which it typically takes for science to have an impact on policy and practice. Participants also spoke about how secure and long-term funding alleviated pressure on team members to be continually applying for external funding, thus freeing up their time to focus their efforts more directly towards stakeholder engagement and impact: *‘It has been a luxury that we are so well funded…we never have to worry about* [applying for grants]*’* (ID 5); *‘…it allows us to try new and different things’* (ID 7). Finally, results show that the allocation of funding should remain flexible and autonomous (i.e. self-managed within the team), to allow for individuals and the broader group to remain responsive to unforeseen opportunities as they arise (e.g. travel to attend unexpected meetings with policy-makers).

Lastly, the interview coding outlined a range of material, practical, political and social features and capacities that can help science to achieve greater impact on policy and practice (Fig 1). Material capacities included the ongoing development of policy-briefs containing specific recommendations that were publicly available, the use of social media platforms (such as Twitter and Facebook) to share information, and an engaging website with lay-person summaries aimed at raising the awareness of key issues among community members (Fig 1). On a practical level, participants also spoke about the need for organisations to provide ‘space’ and ‘time’ for their staff to try new things, to fail and learn, and to pursue independent interests (Fig 1). In terms of political capacity, participants discussed how science can have more impact on policy and practice when focused on ‘hot topics’ that are attracting high levels of social, and thus political, interest: *‘When politicians realise that the public and voters are very engaged in an issue*, *it speeds up their political interest and commitment…this is why we have had success in relation to microplastics*’ (ID 16). Finally, the analysis of interview transcripts identified the need for social capacities (i.e. opportunities to create social networks marked by reciprocity and trust) to be established within research organisations that want to have an impact on policy and practice (Fig 1). Specifically, participants outlined the need for opportunities to have informal face-to-face interactions with decision-makers, so as to build trust and mutual respect for one another.

### Four most important features that enhance impact on policy and practice

In the previous section, we drew upon the experiences of participants to develop a comprehensive list of features that can help research organisations to achieve greater impact on policy and practice. While this list provides guidance for other organisations seeking to build capacity in this regard (Fig 1), most research organisations operate in a highly constrained fiscal environment, and the development and implementation of all of these features is unlikely to be achievable. Further, some of the features are outside of the control of research organisations such as those identified as political capacities. For this reason, it is instructive to identify the most important features, so as to provide a starting point for other research organisations seeking to have more impact on environmental policy and practice.

**Fig 2:**
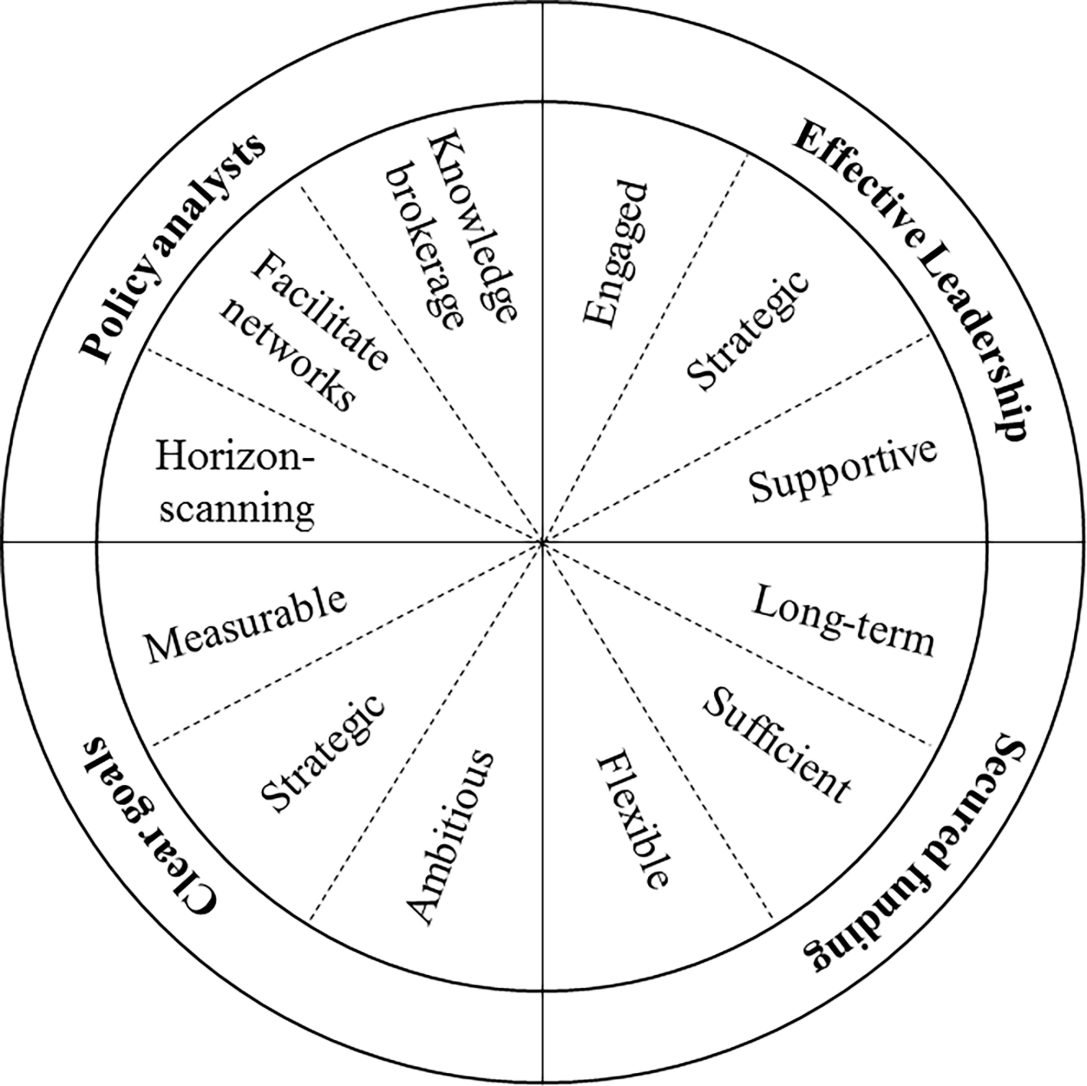
The four most important features of university-based research organisations that increase the impact of environmental science on policy and practice.

Thus, we asked participants to identify the single most important lesson for having impact on policy and practice, based on their experiences working in the Baltic Eye Project (Question 11 in S1 Appendix). Doing so identified four features (Fig 2): i) the inclusion of policy analysts within diverse teams, ii) the establishment of clear goals, iii) the presence of effective leaders, and iv) secured funding. Of these, the inclusion of policy analysts within the team was identified most often (n = 9), with participants outlining a range of benefits conferred to the team through these roles. Specifically, participants spoke about the value of having people that understand local, regional, and international environmental-policy processes for: i) recognising the science needs of policy-makers (i.e. horizon scanning), ii) identifying the most appropriate channel/pathway to influence policy and practice (i.e. matching strategy to context), iii) facilitating knowledge flow among scientists and decision-makers (i.e. knowledge brokerage), iv) training team members in how to most effectively influence policy and practice, and v) facilitating broader and stronger social networks for other teams members (Fig 2). Indeed, the inclusion of policy analysts within the Baltic Eye Project was considered *‘…a real game changer*’ (ID16).

Next, the need for clear goals and effective leadership were also identified as the most critical feature by three participants each (Fig 2). As detailed in the previous section, with respect to goals, participants spoke about the need for them to be ambitious, strategic and measurable. In regards to leadership, participants discussed the importance of having engaged, supportive and strategic leaders who helped to establish clear priorities in support of the broader objectives of the team (Fig 2). Finally, one participant identified secure funding as the most important attribute of research organisations hoping to influence policy (Fig 2).

## Discussion

In the pursuit for decision-making that is informed by scientific evidence, there has been a recent trend towards the implementation of novel institutional structures among research organisations aimed at enhancing the impact of environmental science on policy and practice [17, 21, 22]. Despite these efforts, however, such initiatives are seldom evaluated. Even in cases where evaluations are undertaken, the results are rarely made publicly available [25]. In consequence, there is very little information regarding the organisational features that confer, or limit, success, and there remains a lack of empirically grounded guidance to inform other organisations seeking to develop and implement new institutional approaches in this regard. This study seeks to contribute towards filling this gap, by undertaking an in-depth analysis of the Baltic Eye Project at Stockholm University. In doing so we identified a set of key design features that provides guidance for other organisations seeking to achieve greater impact on environmental policy and practice.

The challenges of identifying non-academic impacts of science–such as social and policy outcomes–are well documented. They arise largely from the ambiguity associated with defining impacts, the non-linear and multi-causal pathways in which impacts occur and the long time-lags that make individual attribution difficult [23, 49–51]. Irrespective, our results suggest that the Baltic Eye Project has had an influence on policy and practice processes relating to the management of the Baltic Sea environment. While the extent to which all of these efforts will translate into actual policies that are implemented and deliver results cannot yet be determined, these findings are encouraging given that the Baltic Eye Project is a young organisation (established in 2013). Indeed, based on evaluations of the UK Research Excellence Framework, it was shown that it typically takes between 3–9 years for impacts to occur [52]. Thus, our results show the speed in which carefully designed research organisations can start to have impact. These findings also highlight the importance of having teams or sections of research organisations focused solely on the science, policy and practice interface. Importantly, they also provide justification for the financial investment in, and mainstreaming of, these types of initiatives.

Study participants also outlined a range of ways in which working in the Baltic Eye Project has impacted them as individuals, for example, via increased job satisfaction. Interestingly, this result contrasts recent perception studies of academics from diverse disciplines that found growing scepticism towards the impact agenda [53, 54]. The sense of job satisfaction reported by participants arose from the learning opportunities associated with working in a diverse team, as well as a sense of personal achievement from observing tangible impacts of science on policy and practice. Indeed, previous studies have shown that while many environmental scientists, particularly early career scientists, are driven by a personal goal of achieving impacts on policy and practice, they are not skilled or trained in how engage with decision-makers, nor understand the complexities of policy processes [8, 55]. This can lead to reduced career satisfaction among early-career scientists, making them less likely to pursue a long-term career in science [56]. Thus, our results also suggest that novel organisational structures like the Baltic Eye Project that focus directly on the interface between science, policy and practice can provide an important new career pathway for early career environmental scientists who may otherwise leave the profession.

Despite the impacts achieved by the Baltic Eye Project to date, participants also outlined a range of barriers that they felt had limited the extent to which they had influenced policy and practice. The most significant of these barriers was a lack of clear goals and direction at the commencement of the Baltic Eye Project. This issue arose partly given the novelty of the Baltic Eye Project, and the lack of available guidance available for them to learn from (which this study seeks to address for future initiatives). The importance of having meaningful goals from the onset of a new initiative is well established across a range of sectors and settings. For example, in business and industry, the development of specific, measurable and strategic goals at the start of a new initiative is considered a key determinant of its overall success [57]. Furthermore, there is ample evidence that successfully meeting goals depends on individuals in leadership positions, who must play an active role (e.g. helping staff to prioritise) in making sure that all team members are consistently working towards their goals in a strategic manner [58, 59]. Similarly, in the environmental sector, the importance of clear goals and effective leaders is also growing [60, 61]. These findings are supported by the participants in this study who also identified clear goals and effective leadership as two of the four most important features of a research organisation seeking to have an impact on environmental policy and practice. However, in a scientific organisation like the Baltic Eye, it is critical to embed these clear goals in a broad and adaptive organisational strategy (or theory of change), which is flexible and allows for adaptation to policy and societal processes.

Our results also highlight the ways in which traditional metrics of academic impact (e.g. number of publications, journal impact factor, h-index, etc.) can limit the extent to which science can have an impact on policy and practice. Indeed, much has been written about the shortcomings and challenges associated with the current metrics of academic impact and the persisting ‘publish or perish’ culture that it creates [62]. For example, it drives researchers to prioritise academic outputs over stakeholder outreach and/or engagement activities, thereby reducing the extent to which new knowledge is incorporated into decision-making processes [28, 63]. It also increases pressure on researchers to work on weekends and evenings, thereby reducing their work-life balance as well as the quality their work [64]. To overcome these challenges research organisations should establish new metrics of impact for non-traditional teams such as the Baltic Eye Project. Specifically, existing reward systems should be broadened to more comprehensively recognise and reward the full suite of activities undertaken by individuals working in teams focused on having impact at the science-policy-practice interface (e.g. developing policy briefs, network development, etc.) [10]. Importantly, these ‘boundary-spanning’ roles are distinct from existing professions in research organisations [11]. Therefore, new and tailored career pathways are also needed to allow the progression, development and retainment of individuals working in these teams [65].

Consistent with the findings of [43] and [44], our results also highlight the importance of having a diversity of skills and experiences within teams focused on improving the relationship between science, policy and practice, and in particular, including people with expertise in processes pertaining to policy and practice. In the case of the Baltic Eye Project these individuals were referred to as policy analysts, and they played a key role in (among others things); (i) policy-scanning to identify policy-windows and the emerging science needs of decision-makers (ii) identifying the most appropriate ways to influence policy or practice, and (iii) establishing networks among team members and external stakeholders. Indeed, it is believed that specialist intermediaries such as these are critical at the interface of science, policy and practice as they are well positioned to proactively identify policy windows [11], which in turn allows research organisations to mobilise their efforts and capitalise quickly on opportunities where scientific evidence could be used to inform decision-making processes [66].

This study also identified a range of individual attributes that team members should possess to be most effective at the interface between science, policy and practice. Of these, the inclusion of individuals with strong and diverse stakeholder networks (or the ability to cultivate strong networks) was considered most important. These findings align with an extensive body of literature that highlights the importance of social networks for environmental management [67, 68], with some authors suggesting that strong social networks can sometimes be more important than the existence of formal institutions for the effective management of environmental assets [69]. This is because the presence of strong and diverse stakeholder networks is thought to facilitate the production, acquisition and transmission of knowledge and information among actors in the network [70].

Our findings also emphasise the importance of having individuals who are resilient, self-motivated and open to new ways of doing things. This can be achieved via two pathways. Firstly, recruitment processes should be sufficiently designed to test for these attributes, for example, via the inclusion of open-ended interview questions. The second, and potentially more promising pathway, is to focus on creating a positive team culture that nurtures and teaches the skills that are necessary to operate effectively at the interface of science, policy and practice. For example, [71] suggests that a greater focus on teamwork training (e.g. via informal team outings, formal teamwork exercises, collaborative team projects, etc.) can enhance interpersonal skills (e.g. social sensitivity, emotional engagement, trust, motivation, etc.) and increase the extent to which a team may solve complex environmental challenges. [72] further suggest that such team building exercises (be they formal or informal) are most likely to enhance team culture when undertaken outside of the place of employment and in ‘inspiring locations’. Thus, by having a strong focus on building a good culture, research organisations can cultivate team members with the necessary skills to have impacts on policy and practice.

Two of the recurrent themes throughout many of the features identified in this study were that of honesty and trust. For example, our results highlight the importance of organisational credibility, which can be achieved through ‘honest brokerage’ [73]. Similarly, participants in our study spoke about the need for honest individuals within the team, so as to allow for the development of trusted relationship with stakeholders. While trust between scientists and decision-makers is often considered as a critical precondition for enhancing the impact of science on policy and practice [74], the exact mechanisms by which trust is formed, maintained and broken in science-policy-practice networks is largely unknown [75–76]. Furthermore, it has also been suggested that too much trust among scientists and decision-makers can lead to adverse environmental outcomes, as well as to reputational damage to individuals and organisations [77]. Accordingly, future research should seek to better understand the mechanisms for developing, maintaining and managing trust at the interface of environmental science, policy and practice.

In undertaking this assessment of the Baltic Eye Project and developing guidance to help other research organisations, we acknowledge that the interface between environmental science, policy and practice is highly context specific across cultures, space and time. Therefore, future studies should seek to understand the suitability and applicability of these findings in other contexts. Doing so will allow for a more representative and robust set of organisational features to be developed, to help research organisations know how to build capacity for greater impact at the interface of science, policy and practice.

Further, while this study has sought to understand the perspectives of people working within the Baltic Eye Project, future evaluations could also seek to include the perspectives of external stakeholders (e.g. relevant policy-makers or other actors). Doing so would allow for a more comprehensive understanding of the enablers and barriers to success at the science-policy-practice interface, and thus allow for the refinement of the principles provided here. However, given the resource intensive nature of qualitative interviews as used in this study, which can limit sample sizes, consideration should be given to other methodological approaches that may be more suitable to understanding the perceptions of broader groups of individuals, such as the use of quantitative surveys [42,46].

Finally, while this study has begun to elucidate the key features that are necessary within university-based boundary organisations to enhance their impact on policy and practice, the ease in which they can be implemented is outside of the scope of our investigation. We suggest, however, that some of the features identified here may challenge entrenched and long-standing traditions and processes within academic research institutions, and the research funders who support their scientific activity [7]. Thus, future research is also needed to understand the broader organisational changes that are required to successfully implement these features [78]. This should include research to understand potential sources of resistance to change [79], and strategies for overcoming them [80, 81].

In conclusion, this study is among the first to empirically evaluate a new research organisational structure aimed at increasing the impact of environmental science on policy and practice. In doing so we have begun to reveal the key features that need to be present within research institutions that want to have impact on policy and practice, and provide guidance for future initiatives to follow. While some of the features identified here may challenge long-standing cultures and processes within research institutions, their implementation will increase the real-world impact of environmental science on policy and practice. Building institutional capacity in this way will be critical if environmental science is to fulfil its societal responsibilities and contribute towards the long-term sustainable management of natural resource underpinning societal well-being and prosperity.

## Supporting information

S1 Appendix Interview guide that formed the basis of data collection. (DOCX)
