# Supplementary material for: Building university-based boundary organisations that facilitate impacts on environmental policy and practice
Source: PLoS One. 2018 Sep 13;13(9):e0203752. doi: 10.1371/journal.pone.0203752 (PMC6136716; doi:10.1371/journal.pone.0203752)
Supplement: S1 Appendix — (DOCX) [file pone.0203752.s001.docx]

The interview guide, as shown here in full, was developed by drawing on and adapting existing published evaluations of knowledge exchange activities, namely [22, 28, 43]. These studies were chosen because, from our knowledge, they represent the most comprehensive and recent evaluations of knowledge exchange in the environmental sector. Further, as these three previous evaluations were undertaken by members of this author team (CC and MR), it was possible to refine the questions based on our previous experiences to suite the specific context of the Baltic Eye Projects. In general, however, questions 1-5 were adapted from the introductory questions used in [28,43]; question 6 was developed by drawing on [22, 28, 43]; questions 7-10 from [28,43]; questions 11-12 from [43]; and questions 13-14 from [28,43].

As detailed in the manuscript, the interview guide was piloted in two stages, firstly among the authorship team and then with two members of the Baltic Eye Project, and refined accordingly. While in some circumstances it is not appropriate to test the interview guide on individuals who are part of the focal research group, given the specific nature of our case study it was important to ensure that the questions were directly understandable and applicable to the specific context. Further, in this instance it was important to test the interview guide in this manner as all interviews were conducted in English (the working language within the Baltic Eye Project), although English was not the native language for most participants. Thus, ensuring clarity in the interview guide was critical.

**Introductory questions**

1. What is your role in [your organisation]?
2. How long have you worked in [your organisation]?

**Understanding the Baltic Eye Project**

1. Please briefly describe the history of the Baltic Eye Project, explaining how it was initiated, why it was designed and how it has evolved over time?
2. What do you understand as the purpose and goals of the Baltic Eye Project?
3. Do you believe that there is an ongoing need for the Baltic Eye Project, why/why not?

**Experience of impacts achieved by the Baltic Eye Project**

1. Considering that a core goal of the Baltic Eye Project is to develop and synthesise knowledge to support evidence-based decisions that improves the Baltic Sea environment, please describe:
   1. The extent to which you feel the Baltic Eye Project has achieved this goal.
      1. Can you think of an explicit example of where knowledge exchange/impact has occurred?
      2. In regards to this specific example, what do you think led to success/failure?
   2. Any other impacts/benefits that have emerged/resulted from the Baltic Eye Project.
      1. Externally to stakeholders (national and international)
      2. Internally within the Baltic Eye, more broadly throughout the Baltic Sea Centre, or Stockholm University.
      3. Personally.

**Factors influencing the effectiveness/impact of the Baltic Eye Project?**

1. What factors do you think led to the Baltic Eye Project achieving these impacts?
2. What, if any, barriers have you experienced that prevented the Baltic Eye Project from achieving these impacts?
   1. How did you try to overcome these barriers?
   2. What could have helped you to overcome these barriers more effectively?
3. Have you experienced any negative or detrimental outcomes associated with the Baltic Eye Project?
   1. Externally to stakeholders (national and international)
   2. Internally within the Baltic Eye or more broadly throughout Baltic Sea Centre or Stockholm University.
   3. Personally.
4. Based on your experiences working in the Baltic Eye Project, have any opportunities for ‘impact’ been missed? If so, what were they and why were they a missed opportunity?

**Building capacity into the future**

1. If there were one lesson you would like other research organisations to learn from your experience in the Baltic Eye Project in terms of supporting evidence-informed decision-making, what would it be?
2. What other generalizable lessons do you think are important for other research organisations to learn from the Baltic Eye Project in terms of supporting evidence-informed decision-making?
3. Based on your experience in the Baltic Eye Project, what capacities/resources do you think are required to build research groups that can effectively support evidence-informed decision-making?

**Conclusion**

1. Now that we have completed the formal component of the interview, are there other important issues that were not covered by our questions, or other relevant insights that you would like to share based on you experience in the Baltic Eye Project?
